# An interview with Mark G. Hans

**DOI:** 10.1590/2176-9451.19.3.026-035.int

**Published:** 2014

**Authors:** Ana Maria Bolognese, Juan Martin Palomo, Kunihiko Miyashita, Lincoln Issamu Nojima, Matilde da Cunha Gonçalves Nojima

**Affiliations:** » DDS, Federal University of Rio Grande do Sul, Brazil. » MSc and Phd in Orthodontics, Federal University of Rio de Janeiro, Brazil. » Postdoctoral in Oral Biology, Northwestern University, Chicago, USA. » Chairman, Department of Orthodontics, Federal University of Rio de Janeiro, Brazil.; » DDS, State University of Ponta Grossa, Brazil. » MSc in Orthodontics, Case Western Reserve University, Cleveland, OH, USA. » Director of Orthodontics Residency - Case Western Reserve University, Cleveland, OH, USA. » Director of Craniofacial Imaging Center - Case Western Reserve University, Cleveland, OH, USA. » Diplomate of the American Board of Orthodontics. » Director of Craniofacial Biology Group - International Association of Dental Research (IADR).; » DDS, School of Dentistry, Nihon University, Tokyo, Japan. » Certificate of Oral Surgeon, Department of Oral Surgery, School of Dentistry, Nihon University, Tokyo, Japan. » Certificate of Orthodontics, Department of Orthodontics, University of California, Los Angeles, USA. » PhD, Department of Anatomy, School of Dentistry, Nihon University, Tokyo, Japan. » Visiting Professor, University of California, Los Angeles, USA. » Adjunct Professor, Case Western Reserve University, Cleveland, OH, USA. » Member of the Bolton-Brush Growth Study Center, Case Western Reserve University, Cleveland, OH, USA. » Director at the Foundation of Maxillo-Facial-Research, Tokyo, Japan.; » DDS, University of Passo Fundo, Brazil. » MSc and Phd in Orthodontics, Federal University of Rio de Janeiro, Brazil. » Postdoctoral in Orthodontics, Case Western Reserve University, Cleveland, OH, USA. Capes Scholarship 0906/11-6. » Associate Professor, Department of Orthodontics, Federal University of Rio de Janeiro, Brazil. » Diplomate, Brazilian Board of Orthodontics.; » DDS, Federal University of Rio de Janeiro, Brazil. » MSc and Phd in Orthodontics, Federal University of Rio de Janeiro, Brazil. » Postdoctoral in Orthodontics, Case Western Reserve University, Cleveland, OH, USA. Capes Scholarship 1540/11-4. » Associate Professor, Department of Orthodontics, Federal University of Rio de Janeiro, Brazil.

## Abstract

It is a great honor to conduct an interview with Professor Mark G. Hans, after following his outstanding work ahead of the Bolton-Brush Growth Study Center and the Department of Orthodontics at the prestigious Case Western Reserve School of Dental Medicine (CWRU) in Cleveland, Ohio. Born in Berea, Ohio, Professor Mark Hans attended Yale University in New Haven, CT, and earned his Bachelor of Science Degree in Chemistry. Upon graduation, Dr. Hans received his DDS and Masters Degree of Science in Dentistry with specialty certification in Orthodontics at Case Western Reserve University. During his education, Dr. Hans' Master's Thesis won the Harry Sicher Award for Best Research by an Orthodontic Student and being granted a Presidential Teaching Fellowship. As one of the youngest doctors ever certified by the American Board of Orthodontics, Dr. Hans continues to maintain his board certification. He has worked through academics on a variety of research interests, that includes the demographics of orthodontic practice, digital radiographic data, dental and craniofacial genetics, as obstructive sleep apnea syndrome, with selected publications in these fields. One of his noteworthy contributions to the orthodontic literature came along with Dr. Donald Enlow on the pages of "Essentials of Facial Growth", being reference on the study of craniofacial growth and development. Dr. Mark Hans's academic career is linked to CWRU, recognized as the renowned birthplace of research on craniofacial growth and development, where the classic Bolton-Brush Growth Study was historically set. Today, Dr. Hans is the Director of The Bolton-Brush Growth Study Center, performing, with great skill and dedication, the handling of the larger longitudinal sample of bone growth study. He is Associate Dean for Graduate Studies, Professor and Chairman of the Department of Orthodontics, working in clinical and theoretical activities with students of the Undergraduate Course from the School of Dental Medicine and residents in the Department of Orthodontics at CWRU. Part of his clinical practice at the university is devoted to the treatment of craniofacial anomalies and to special needs patients. Prof. Mark Hans has been wisely conducting the Joint Cephalometric Experts Group (JCEG) since 2008, held at the School of Dental Medicine (CWRU). He coordinates a team composed of American, Asian, Brazilian and European researchers and clinicians, working on the transition from 2D cephalometrics to 3D cone beam imaging as well as 3D models for diagnosis, treatment planning and assessment of orthodontic outcomes. Dr. Hans travels to different countries to give lectures on his fields of interest. Besides, he still maintains a clinical orthodontic practice at his private office. In every respect, Dr. Hans coordinates all activities with particular skill and performance. Married to Susan, they have two sons, Thomas and Jack and one daughter, Sarah and he enjoys playing jazz guitar for family and friends.

Matilde da Cunha Gonçalves Nojima

**Figure:**
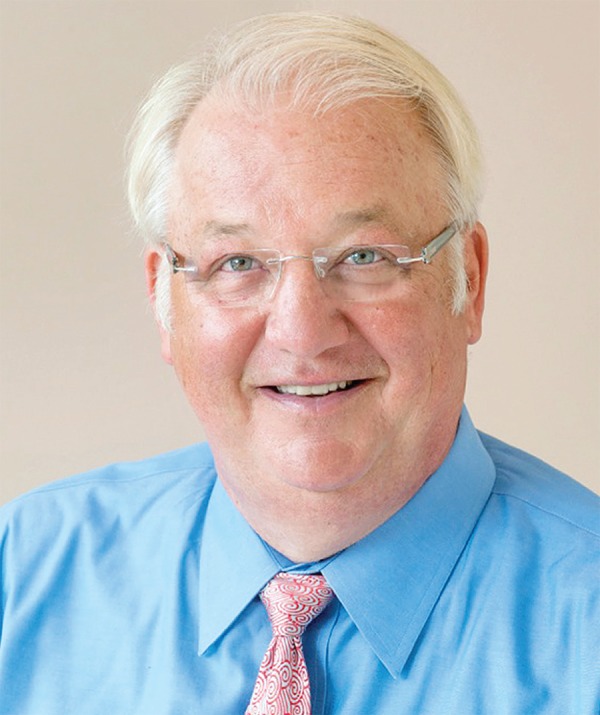


## 1. As director of the Bolton-Brush Growth Study Center, could you tell us some points about this renowned research center? (Lincoln Nojima)

Located on third floor of the Bolton Dental Building, the Bolton-Brush Growth Study Center houses the world's largest longitudinal radiographic collections of cranial and post cranial skeleton. The Bolton Study was started in 1930 by the inventor of the cephalostat, B. Holly Broadbent, with the goal of increasing our understanding of the normal growth of the human face. A total of 4,309 subjects were enrolled. Lateral and frontal cephalograms as well as hand wrist radiographs and dental study casts were taken yearly on these children, usually on or around their birthday. Under the direction of T. Wingate Todd, the Brush Study began at the same time with the goal of radiographically documenting the normal growth and development of the appendicular skeleton. All of Bolton subjects were also enrolled in the Brush Study. The Brush Study ended in 1950 and the Bolton study ended in 2001. To be part of the Bolton-Brush subject population, individuals had to be in excellent health and free from any major illness or infirmity. Often, these children were enrolled because they had won "Healthy Child" contests at their schools. Landmark publications resulted from these legendary studies. Some examples are the Greulich and Pyle Hand Wrist Atlas used by pediatricians around the world to assess skeletal development in growing children, the Bolton Standards for Dentofacial Growth and Development used by craniofacial practitioners to assess facial proportions and skeletal balance, as well as the classic work by Rolf Behrents on Adult Craniofacial Growth and Development.

Although we stopped enrolling new subjects in these studies, the collections are still used by researchers from around the globe to answer important questions pertaining to human health. One of the main activities for center staff is to convert the fragile radiographs to digital format. A small portion of the Bolton Collection is available online by visiting the American Association of Orthodontist Legacy Collection website: http://www.aaoflegacycollection.org/aaof_collection.html?id=CASEBolton

Many more radiographs are available in digital format and can be accessed at the center or by ordering digital copies of the radiographs. A complete index to the radiographs in the Bolton and Brush collections is available by sending an email to mark.hans@case.edu with the subject line "Bolton-Brush Index". The index is in Filemaker Pro format and a demo version of the Filemaker Pro program can be downloaded for free from the Filemaker Pro website.

## 2. In your opinion, which theories of Facial Growth should be taught in an orthodontic program? (Juan Martin Palomo)

**Figure 1:**
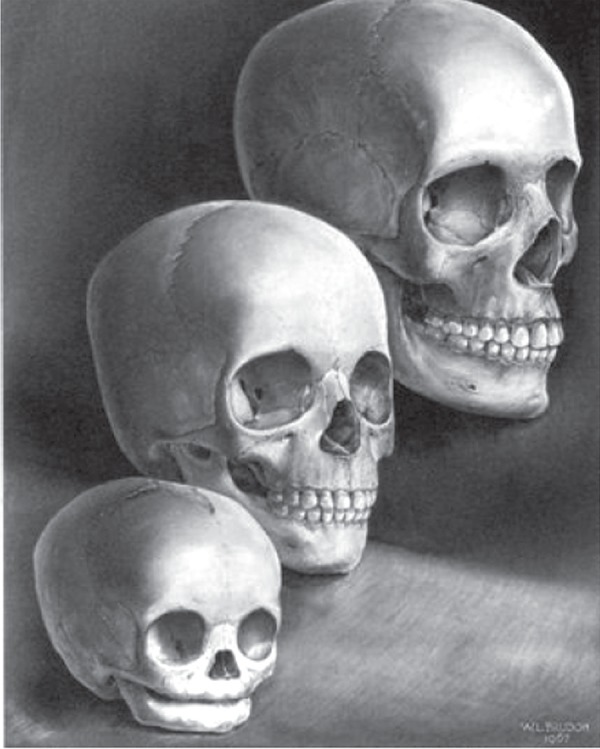
Morphologic changes of the face. The infant and young child are characterized by a wide-appearing face but vertically short. During later childhood and into adolescence, vertical nasal enlargement keeps pace with growing body and lung size, dental and other oral components have approached adult sizes and configuration. Overall, the early wide face has become altered in proportion by the later vertical changes. (In Enlow, D. H. and Hans, M. G.: Essentials of Facial Growth. 2^nd^ ed. Ann Arbor: Needham Press, 2008. Courtesy of William L. Brudon. From Enlow, D. H.: The Human Face. New York, Harper & Row, 1968, with permission).

In my opinion, we do not understand the switches that turn bone growth on and off. However, the morphologic changes that occur as a result of the growth process have been well described (Fig 1). Therefore, orthodontic education should focus on giving students a thorough understanding of morphologic changes that occur as well as the biological processes that result in those changes. For example, orthodontic programs must teach students about bone remodeling and displacement. Remodeling activity includes the deposition of new bone on both periosteal and endosteal surfaces by osteoblasts concomitant with the resorption of bone on these surfaces mediated by osteoclasts. The areas of bone deposition and resorption were mapped for the human face as part of the life work of Donald H. Enlow, PhD. Dr. Enlow was my predecessor as department chair at CWRU and I was very fortunate when he asked me to coauthor the textbook Essentials of Facial Growth in 1996. Now, in its second edition, Essentials describes in detail, the remodeling process. As second equally important concept is that of bony displacement. Displacement is the movement in space of an entire bone en masse. All morphologic changes we see in the bony skeleton are the result of these two basic processes. And since all orthodontic and orthopedic treatments must affect changes in one or both of these processes, they must be taught in all orthodontic training programs.

In terms of theories of facial growth, I think it is important for students to know something about Moss' Functional Matrix hypothesis as well as Scott's Nasal Septum Theory. Each of these theories adds something of value to students understanding of facial growth. In the case of the functional matrix hypothesis, it leads the student to think about the relationship between hard and soft tissues of the head and neck. The theory is particularly strong when applied to the growth of the neurocranium. In this area, growth of brain tissue clearly paces enlargement of the flat bones of the skull. As you move into the area of the cranial base, Scott's cartilage theory becomes attractive because of the ability of cartilage to grow interstitially and the histologic similarity between synchondrosis and epiphyseal growth plates. As we approach areas closer to the occlusal plane, things get more confusing. For example, what is the most logical theory regarding the tissue separating force that drives midfacial growth? The nasal septal cartilage sits in the midline and the septopremaxillary ligament attaches to the anterior nasal spine of the maxilla allowing expansile growth of the cartilaginous septum to pull the maxilla downward and forward. This is consistent with the observed displacement of the maxilla downward and forward. The functional matrix theory holds that it is respiration and in particular nasal respiration that drives growth of the midface downward and forward. It is most likely that both of these processes (i.e. septal growth and respiration) influence maxillary development, and further, that a deficiency in one driving force is likely compensated by an excess in the other. That there exists a certain amount of redundancy in critical growth systems seems plausible. However, the complexity of human maxillary growth will likely not allow the relative contributions of the functional matrix compared to the nasal septum to be tested by hypothesis driven research methods. Finally, neither theory provides a compelling rationale for mandibular growth. Clearly, the mandibular condyle does not function as a locus of control. Rather, the most important role for this cartilage is in establishing and maintaining the integrity of the temporomandibular joint. It is also difficult to comprehend how mastication and/or deglutition influence mandibular growth. I would say that both theories fail to provide a compelling argument for the driving force behind the downward and forward displacement of the mandible.

## 3. How do you focus on the importance of knowledge on Craniofacial Biology for orthodontic treatment planning and achievement of better treatment outcomes? (Matilde Nojima)

The goal of orthodontic treatment should be to transform one stable system (malocclusion) into another stable system (normal occlusion) using a combination of growth modification and dental movement in the growing patient and a combination of orthognathic surgery and dental movement in non-growing patients. So, in my mind, better treatment outcomes mean more stable outcomes. How can we best achieve this goal? I think we need to consider several factors. First, where the cause of the malocclusion can be identified, we need to treat the cause first. The best example of this strategy is with a child that sucks his thumb. We need to stop the habit first, then if the malocclusion does not spontaneously improve, we need to intervene to treat the effect of the thumb habit. Stability will be achieved since the cause of the malocclusion has been addressed. Unfortunately, in most malocclusions the cause is less obvious. Most of us would agree that malocclusions are the result of both intrinsic (i.e. genetic) and extrinsic (i.e. environment) factors. I don't think anyone is considering gene therapy for malocclusion so that leaves us with modifying extrinsic factors and treating the net effect of the genetic factors rather than the genetic cause. Orthodontists need to be very effective at moving teeth in all three dimensions (vertical, lateral, and horizontal) within the alveolar process. We need to pay attention to tooth movements and control the movements. To do this effectively, we should know the normal way teeth move during growth and development. We are all familiar with the term eruption of teeth. I want to clarify an important difference between eruption of teeth and drift of teeth. Eruption is a biologic process whereby the tooth moves towards the occlusal plane until it contacts its opposing tooth and is said to be "in occlusion". Once a tooth is in occlusion, it is finished with the eruption process. However, the face continues to grow downward and forward and to maintain this occlusion of the teeth, they must drift towards the occlusal plane. This means maxillary teeth drift inferiorly and mandibular teeth drift superiorly. In addition to the vertical drift of teeth, the teeth drift mesially with normal growth and development. Thus, drift of teeth naturally occurs toward the occlusal plane and mesially. So, what does this mean for the average orthodontist and their patients? We all know it is easier to move teeth in the direction they naturally want to go. So, if you do not pay any attention to your treatment mechanics, you will naturally accelerate the movement of teeth toward the occlusal plane and mesially. This means that when you are finished with your treatment, the face will be longer (vertical dimension will be increased), and the teeth will be more forward in the face (i.e. more bimaxillary protrusive). If these movements are outside of the physiologic boundaries of stability, then you will have created an unstable result. Not what we are looking for. So, to achieve better treatment outcomes, you need to pay attention to your mechanics if you do not want a longer face or a more protrusive denture. The skilled orthodontist can control the teeth in all three dimensions so it is critical that we do so.

## 4. In order to better understand growth and development of the face, do you believe that establishing VTOs on growing patients can be helpful? If so, which method do you recommend us to use? (Kunihiko Miyashita)

To be an excellent orthodontist, you need to have two goals in mind. First, the alignment of the teeth and second the placement of the teeth within the face. The alignment of teeth is almost always dictated by the contact points of adjacent teeth. This results in little disagreement among dentists as to the ideal position of the teeth relative to each other. Likewise, there is general agreement among dentists about the proper occlusion of the teeth, that is, axial loading of posterior teeth with anterior guidance provided by the cuspids and incisors. However, there is not universal agreement as to the ideal place for the teeth within the face. It is in this area that I believe a VTO is helpful. A VTO gives the orthodontist a target to shoot for and he or she should aim for the bull's eye. Without a target you cannot measure the accuracy of treatment plan for the position of teeth within the face.

So, I believe you should have a goal for the position of the dentition within the face. I prefer to start my VTO by asking the question, "Where should the upper central incisor be located in this patient's face?" There are several ways to determine this position. I prefer to use the Bolton Standards as a guide for upper incisor location. In addition, I confirm the use of the Bolton Standard position by using a Nasion Vertical and placing the maxillary central incisor 5 mm in front of the line and vertically about 2-3 mm below the inferior border of the upper lip in the relaxed state. Once I have established the position of the upper incisor, I then decide on how best to establish anterior guidance and an acceptable interincisal angle. In terms of growth prediction, I prefer to use a mean change expansion as described by Johnston et al.^1^ In general, this method assumes that the maxilla will grow 1 mm forward at A Point per year and 1 mm vertically at ANS per year. Mandibular growth will exceed maxillary growth by 1 mm per year in both vertical and horizontal directions.

## 5. What are the biological indicators that should be observed in order to predict the amount of future growth in a specific bone of the face? (Ana Maria Bolognese)

In general I think that the Greulich and Pyle Hand Wrist Atlas is useful to estimate skeletal age and predict standing height. However, it has been well documented that facial growth cannot be predicted using skeletal maturity as an indicator. So, the best assumption for growth of the facial bones - i.e. the maxilla and mandible - is to assume that in the absence of treatment, mandibular growth will exceed maxillary growth by about 1 mm per year. In terms of estimating the amount of growth, one can use chronological age, hormonal indicators of maturity (acne, onset of menstruation in girls, facial hair in boys) to help estimate the amount of growth potential remaining. However, I still believe that the only way to determine when facial growth has slowed is to use serial cephalometric radiographs taken a minimum of 6 months apart. When the two films shows less than 0.5 mm of change over a six month period prior, then the clinician can safely assume adolescent facial growth has been completed.

## 6. What are your thoughts on the best timing to treat skeletal disharmonies as Class III malocclusions? (Matilde Nojima)

I am glad you asked this question because although there are three treatment options for Class III correction: orthopedic modification of facial growth, orthodontic camouflage, and orthognathic surgery; the age of the patient often dictates the best treatment strategy. The orthodontic literature clearly indicates that full orthodontic treatment increases mandibular growth. I call this non-specific stimulation of mandibular growth the "fertilizer effect". By this, I mean that no matter what we do with our mechanics, we tend to increase the amount of mandibular growth a patient has during treatment. For treatment of Class II malocclusion, the fertilizer effect is beneficial, but in Class III treatment it is not helpful. The fertilizer effect is greatest during periods of rapid facial growth. So, my first rule for treating Class III is to try to start treatment either before or after pubertal growth spurt. This is fairly easy to achieve for our orthopedic procedures. In terms of orthopedic intervention for Class III, I think data on the success of face mask protraction to stimulate maxillary growth is much stronger than data on successful restraint of mandibular growth using a chin cup. Therefore, I recommend the use of protraction facemask therapy for developing Class III malocclusions. It is my opinion that this type of therapy can be done at any time before fusion of the maxillary suture system. However, I think the optimal time for treatment is after eruption of the maxillary and mandibular central incisors. I prefer to wait until this time for two reasons. First, treatment can still be completed well before the onset of puberty, and second, you can establish normal overbite and overjet of the permanent incisors so we do not need to have any sort of retention appliance used after the anterior crossbite is corrected. If you correct Class III any sooner, you have the problem of retaining correction during the transition of the incisor dentition. It is important to me that there be contact of natural dentition during the post treatment period. I think proprioceptive feedback during function is an important factor in the success of early Class III treatment. Ideally, if I treat a patient early for Class III, I like to wait to begin full fixed orthodontic treatment until after the pubertal growth spurt is complete.

If you miss the orthopedic treatment window, then I think it is best to wait to begin full fixed treatment until after the pubertal growth spurt. I think this is best because at this point you have only two options remaining, camouflage or surgery. And, in one case (camouflage) you will be adding dental compensations to establish the best possible occlusion while in the other (surgery) you will be removing dental compensations to allow correction for skeletal disharmony. It is impossible for the orthodontist to do both at the same time. So, you are forced to choose. And delaying the choice as long as possible gives you the best chance to make the correct decision between these two options.

## 7. What are your views on growth modification through the use of functional appliances? (Juan Martin Palomo)

I believe the data on modification of maxillary growth both forward using a protraction facemask or backward using cervical pull headgear is compelling and I recommend these treatment options to my younger patients who would benefit from such therapy. I recommend that these orthopedic devices be worn only at night because we know that humans only grow at night and that teeth only erupt at night. There seem to be two major factors that predict treatment success. The first is patient compliance. To see any effect with the headgear, it must be worn almost every night. Since orthopedic treatment seeks to influence facial growth, we have to allow time for growth to occur. This means that a minimum of 6 months of compliant wear must be achieved before you can assess the second factor that influences treatment success. That second factor is the genetic susceptibility of the patient to growth modification, i.e. is the patient a "responder" or a "non-responder" to orthopedic therapy? I think most orthodontists tend to think all non-responders are non-compliant, but I disagree. I think you can get most patients to comply with treatment for a few months. The ones that see results are encouraged and continue to wear their device at night, the ones that do not respond get discouraged and stop wearing the device. I never blame the patient for not responding, in most cases it is not their fault. As my friend Gerry Samson says: "You can't ask them to take another dip in the gene pool". It would be great if we could find a way to determine responders from non-responders without having to wait 3-6 months, but I do not see that as an option in the near future.

## 8. Since you are a great expert on craniofacial growth, what is your opinion on the historical debate between functional appliances and headgear traction for the correction of Class II malocclusions? (Lincoln Nojima)

I take an approach to the use of headgear traction in the correction of Class II that is very similar to Dr. Robert Ricketts. I like to use a cervical pull facebow in combination with a lower utility arch. The headgear is effective in correcting maxillary skeletal prognathism and maxillary dental protrusion, and the utility arch uncouples the upper and lower anterior teeth by intrusion of lower incisors. Ricketts's theory, and I agree, was that by uncoupling the anterior teeth you allow the mandible to be displaced downward and forward. Since the effect of orthodontic treatment on mandibular growth is non-specific and highly variable, I do not find a big difference in mandibular growth response between functional appliances that advance the mandible and headgear/utility arch mechanics. And, since headgear is a fixed device, it is much more effective at addressing maxillary protrusions that often accompany Class II malocclusions.

By the way, I use the cervical pull headgear exclusively for facebow type headgear traction. I find the posterior high pull headgear not as effective as an anterior J-Hook headgear in controlling vertical. Plus, you need to add the Transpalatal Arch to the Posterior high pull to negate the buccal rolling of molars. I used to apply straight pull facebows but found that patient compliance was much better with the simpler cervical pull. And, since I do not use facebow headgear in high angle cases, the small difference between the angle of pull for straight pull and cervical was not clinically significant.

## 9.Do you think that using temporary anchorage devices (TAD) can help us control craniofacial growth patterns? (Kunihiko Miyashita)

I think that TADs can help control vertical dental drift in growing patients. As mentioned earlier, vertical drift of dentition occurs towards the occlusal plane. So, controlling this natural movement could be helpful in patients with increased lower vertical facial height. Of course, to achieve this goal it would be necessary to control both the inferior drift of the maxillary buccal segments, as well as the superior drift of the mandibular buccal segments. In addition, because we are trying to limit vertical facial development by modifying the dentition and alveolus, these mechanics would likely need to be continued until vertical facial growth was completed. This type of growth modification will face the same challenges as we faced when we used chin cups to limit mandibular growth, i.e. achieving long-term stability will require a long-term retention strategy. I have used miniplates to intrude posterior segments in several patients including one that we published in the Journal of Plastic Surgery. TADs are an exciting addition to our mechanical systems and their effective use will require orthodontists to apply bone biology in their TAD placement planning. For example, long-term placement of a TAD in an area of bone that is undergoing bone resorption as part of remodeling process will likely fail. Whereas, placement of a TAD in an area of natural bone deposition has a higher chance of success based on biology. Applying this concept to vertical control of dental drift would mean that maxillary TADs should be placed in the palate, and not on the buccal surface. In the mandible, TADs could be placed on the buccal cortices adjacent to the molars. TADs placed in alveolar bone are likely to fail sooner than those placed in cortical bone. Since you need long-term TAD success to modify craniofacial growth, the resorptive and depository remodelling patterns are important to understand.

## 10. Is there any different clinical response in the sutural tissues of growing patients using mini-plates or headgear traction considering growth and displacement of facial bones? (Ana Maria Bolognese)

Absolutely!!! This is one of the biggest areas of confusion regarding the use of miniplates compared to using headgear. The goal of applying orthopedic forces to the growing craniofacial complex is to change the displacement and remodelling of the bones. For example, cervical pull headgear can be used in a variety of ways to change facial growth depending on how the force is applied. Dr. Andy Haas, Robert Ricketts and others have documented the effects of cervical pull headgear applied solely to maxillary first molars. When the headgear is attached only to maxillary first molars you are using the teeth via the periodontal ligament (PDL) as a transducer to send mechanical signals to the periosteum and sutural systems. The sutural system can be influenced by this mechanical system including the intermaxillary suture, the circummaxillary suture system, and, to a lesser extent, the circumfacial sutures. Cervical headgear applied to maxillary first molars combined with a lower utility arch to intrude the lower incisors can also influence mandibular growth. The most logical explanation of the effect of cervical headgear on mandibular growth is that by disengaging the dentition with the intrusion arch, the mandible outgrows the nasomaxillary complex. In contrast to the biological impact of orthopedic force application, miniplates are just devices that can be rigidly attached to bone. There is no biologic rationale to think that miniplates are anything like headgear.

## 11. Considering the important concepts included in your classic "Essentials of Facial Growth", how do you feel about research on craniofacial growth and airway? And how such information can be correlated to clinical intervention? (Matilde Nojima)

Probable, one of the most classic experiments that demonstrated the impact of forced oral respiration on facial growth was conducted by Egil Harvold when he plugged the noses of growing rhesus monkeys. What everyone remembers about this experiment is that Harvold was able to cause open bite malocclusions in these monkeys. And these open bite malocclusions were characterized by increased lower vertical facial height, maxillary transverse deficiency and dental crowding. What most people forget is that not all of Harvold's monkeys developed malocclusions. This variability in response to airway obstruction has not been talked about very much in our literature. In my opinion, if we have such variability in a genetically homogeneous population of experimental animals I would expect even greater variation in human populations. And, this is in fact what we have found with any large study on the impact of airway on facial growth. It is impossible to show a simple cause and effect relationship between mouth breathing and malocclusion. It makes sense that there should be some influence since the roof of the mouth and the floor of the nose are the same bone, but I do not think we will ever be able to prove such a relationship. In terms of clinical intervention, I think we do know that rapid palatal expansion reduces nasal airway resistance. And, we know that moving the mandible forward with functional appliances increases the oral pharyngeal airway. These are anatomic facts. Therefore, if you have a patient that has nasal obstruction and is a mouth breather you could consider palatal expansion as one mode of treatment. If the pediatrician asks whether removal of adenoid tissue would be beneficial for facial growth I would answer the following way: If the child needs to have adenoid tissue removed for medical reasons (i.e. recurrent infection), then I would support the operation and indicate that there could be a positive effect on facial development. In contrast, if a pediatrician asks me if I would recommend removal of adenoid tissue for improving facial growth I would say that the evidence is not strong enough to support such a recommendation from the orthodontist.

## 12. What changes can be achieved, after growth, to correct the morphology of the nasomaxillary complex in cases of open bite and mouth breathers ? (Ana Maria Bolognese)

After the pubertal growth spurt and adolescent growth has completed, I think that the only changes in the morphology of the nasomaxillary complex that can be reliably achieved involve remodeling of the alveolar processes that accompanies tooth movement. However, these changes can be significant, especially when permanent teeth are removed. We published several interesting studies on the influence of extraction of permanent teeth on vertical facial growth. We found that after growth, the extrusive effects of orthodontic treatment are minimal. This really helps in the correction of anterior openbite. The clinician does not have to worry as much about molar extrusion in non-growing patients. We were able to show that correction of openbite was achieved primarily by uprighting upper and lower incisors. This was true for extraction of first bicuspids as well for extraction of permanent first molars. I think the tendency for treating without extraction of permanent teeth has severely limited what we can achieve for our patients after growth. For the non-growing patient, the need for extraction of permanent teeth must be carefully evaluated.

## 13. How do you address the relationship between Craniofacial Growth and stability of orthodontic treatment outcomes? (Lincoln Nojima)

This is an interesting question, especially as it pertains to craniofacial growth that occurs after orthodontic treatment. During treatment, the orthodontist is constantly monitoring craniofacial growth, treatment response, and the progress towards completion of therapy. Treatment decision can be made on a monthly basis to compensate or decompensate the dentition in response to craniofacial growth. Once the treatment goals of dental alignment, anterior guidance, axial loading of posterior teeth, proper smile arch, pleasing smile, etc., have been achieved and braces are removed, the game changes. Now, the patient no longer has the skilled orthodontist to help maintain equilibrium among all of components of the craniofacial complex that are involved in maintaining the dental occlusion. Although there are no easy answers to this dilemma, I can offer one suggestion. Do not try to finish all of your cases with centric relation (the ligamentous position of the mandibular condyle in the glenoid fossa) coincident with the position of the condyle in the glenoid fossa dictated by the maximum intercuspation of the teeth. Allow at least 1-2 mm of difference between these two condylar positions. That way, if you get a couple millimeters of late mandibular growth, a change in CR-MIC relationship can occur and compensate for this growth, thereby keeping teeth in proper occlusion.

## 14. In a constantly changing craniofacial complex, what are your views on the changes that occur during adulthood, the appearance of late dental misalignment and the concept of retainers for life? (Juan Martin Palomo)

The publications from the University of Washington group on long-term stability of treated malocclusions in the mid 1980's coupled with the natural bias of doctors and patients against extraction of teeth, caused a monumental paradigm shift in Orthodontics. Prior to these publications, orthodontists considered stability of orthodontic correction to be one of the main goals of treatment. And to achieve this goal, the removal of permanent teeth was often prescribed so that expansion of dental arches in the anterior and lateral dimensions could be avoided. After these publications, lifetime retention was de rigueur for all patients. I think that this change in our approach to stability is very dangerous to our profession. The main reason for my thinking is that if we give up on stability as a treatment goal, we run the risk of diminishing the specialty to the level of a cosmetic rather than a functional service. This, in turn, will make it much easier for the public to view orthodontic treatment as a commodity. When the public purchases a commodity, they focus on value. If stability is not part of the value equation, we are left with price and treatment time. Patients will assume that quality of treatment results is equal among providers. Our specialty is much more than aligning teeth. Fit and function as well as lifetime dental health should be important goals of treatment. Making beautiful smiles is fine, but we cannot do so at the expense of dental health.

My main concern about lifetime retention is that there is a real possibility that significant changes in the supporting periodontium could result if teeth retained for a lifetime in an unstable position. We know from our studies of bone biology that under pressure bone resorbs. So, if an orthodontist moves teeth beyond their physiologic boundary, pathology will ensue. The critical question is "What are those boundaries?" Identifying these boundaries should be the focus of orthodontic research for the coming decade. Until we have a better idea of the limits of treatment, we should be wary of lifetime retention. I think a more biologic approach would be to tell our patients that we can produce an orthodontic treatment result as good as someone who was born with straight teeth. However, we know from the Bolton Brush Studies, that naturally straight teeth do not stay that way for a lifetime. The idea that a single orthodontic intervention will lead to a lifetime of perfectly straight teeth is not realistic. We should inform patients that additional treatment may be needed in the future due to naturally occurring growth of the adult craniofacial skeleton.

## 15. Which bone is your favorite? And why? (Kunihiko Miyashita)

**Figure 2:**
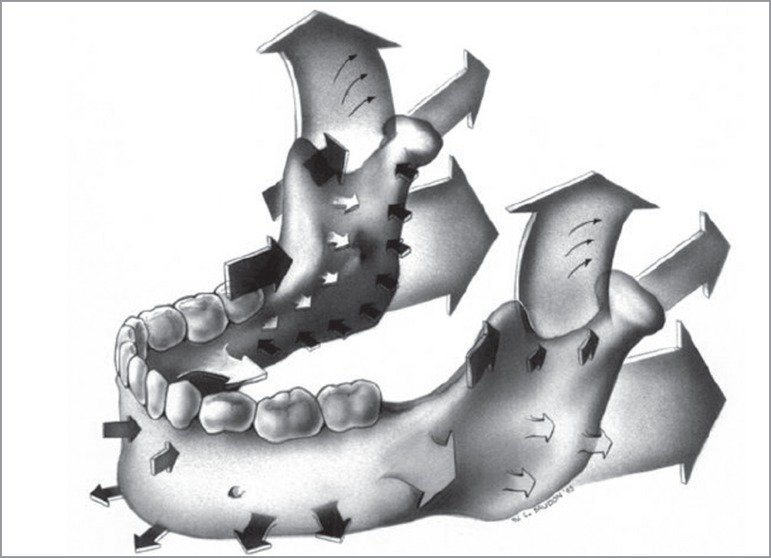
Summary diagram of the growth of the mandible. Growth directions involving periosteal resorption are indicated by arrows pointing into the bone surface, and growth directions involving periosteal deposition are represented by arrows pointing out of the bone surface. (In Enlow, D. H. and Hans, M. G.: Essentials of Facial Growth. 2^nd^ed. Ann Arbor: Needham Press, 2008. From Enlow, D. H. and D. B. Harris: A study of the postnatal growth of the human mandible. Am. J. Orthod. 50 (25), 1964, with permission.)

Without question, the mandible is my favorite bone in the craniofacial regions. My attraction to the study of the mandible is based on three factors. First, the historic orthodontic controversies involving this bone. These begin with the myth of the condylar cartilage as a growth center that magically determined the size and shape of this complex bone. This was followed by the functional appliance craze in the United States with the outrageous claims of mandibular protrusive devices being capable of stimulating mandibular corpus growth in the six to seven millimeter range. Then, came the false claims that malocclusions caused temporomandibular joint disease. And finally, the most recent controversy over the role of the size and shape of the mandible in Obstructive Sleep Apnea Syndrome. The second reason I am fond of the mandible is its anatomic complexity (Fig 2). When I teach students about the mandible I like to divide the bone into five areas based on function. Area one, the condyle with the primary function of articulation. Area two, the coronoid process, primary function, attachment of the temporalis muscle. Area three, the corpus, primary function to connect the right and left halves for the mandible as a rigid strut. Area four, the alveolus, primary function to support the dentition. Area five, the ramus, primary functiont to provide compensations in both the vertical and horizontal dimensions to insure occlusion of molar teeth within 6 mm among the entire human race. Finally, I like the mandible because we have much to learn about the control processes involved in its growth. Right now, I like to say to my students that all orthodontic/orthopedic treatments of growing patients increase mandibular growth a little bit, I refer to this non-specific stimulation of mandibular growth as the "fertilizer effect".
